# Commentary: “The Role of T3 Surface Molecules in the Activation of Human Cells: A Two-Stimulus Requirement for IL-2 Production Reflects Events Occurring at a Pretranslational Level”

**DOI:** 10.3389/fimmu.2015.00163

**Published:** 2015-04-21

**Authors:** Arthur Weiss, John D. Stobo

**Affiliations:** ^1^Department of Medicine, Rosalind Russell-Ephraim P. Engleman Rheumatology Research Center, Howard Hughes Medical Institute, University of California, San Francisco, CA, USA; ^2^Office of the President, University of California, Oakland, CA, USA

**Keywords:** T cell, interleukin 2, Jurkat cells, immunology, T3 surface molecules

In 1982, identifying the T cells antigen receptor was still the elusive “Holy Grail” of immunology. However, the ability to use monoclonal antibodies (mAbs) to identify and characterize molecules on lymphocytes, coupled with the then recent ability to grow long-term antigen-specific T cell clones, provided a strategy to identify the TCR by isolating clone-specific mAbs. We decided to take on this ambitious, but exciting project.

We set out to grow allo-reactive human T cell clones with different specificities in order to use one clone as an immunogen and other clones with different specificities as controls. To grow human T cell clones, a source of growth factors to maintain long-term T cell clones was needed and the recently identified interleukin-2 (IL-2) was the best candidate. However, *human* T cells required *human* IL-2 for their propagation and it was going to be cumbersome to stimulate large numbers of human peripheral blood T cells for a source of the growth factor. The IL-2 gene had only recently been cloned and recombinant IL-2 was not available. However, work from Gillis and Watson described a human acute lymphoblastic leukemic T cell line called Jurkat that could be stimulated with phytohemagglutinin (PHA), a mitogenic plant lectin reactive with carbohydrates, to produce IL-2 (1). Stimulating large numbers of Jurkat cells to produce IL-2 for T cell cloning purposes offered a simple solution to our dilemma. We were able to obtain the Jurkat line from Kendall Smith and it seemed our problem was solved. PHA could stimulate the line to produce moderate amounts of bioactive IL-2 and the amount that it produced could be boosted by the addition of the tumor promoter, phorbol myristate acetate (PMA).

Just as we were getting started on the project, we were dealt a crushing blow by a paper from Meuer et al., who used T cell clone specific mAbs to convincingly identify the TCR (2). They found a clone specific αβ heterodimer on a human T cell clone. The identification of the αβ heterodimer as the TCR was consistent with a tumor-specific structure that had previously been identified by Jim Allison’s group, who had speculated that the tumor-specific heterodimer might potentially represent the TCR (3). Importantly, Meuer et al. also suggested that the clone-specific heterodimer that they identified was associated with the T3 (later named CD3) complex (2), whose expression was previously linked to antigen-specific recognition (4). It had been known for a few years that mAbs against T3, like PHA, were mitogenic and could substitute for antigen in inducing T cell activation (5, 6). As we regrouped, it occurred to us that since Jurkat cells could be activated by PHA, Jurkat might express T3 and even an antigen receptor. Indeed, we found that Jurkat expressed T3 antigens. In parallel studies, we went on to make clone specific mAbs to the Jurkat αβ heterodimer, including the IgM C305 which is a Vβ8 specific mAb commonly used in Jurkat studies today (7).

We considered the possibility that Jurkat might be stimulated via its TCR-T3 complex, but stimulation of the cell with only anti-T3 mAb resulted in no detectable secreted IL-2, as assessed by bioassay (note IL-2 was detected by bioassay using the IL-2 dependent clone CTLL-20). However, we found that the addition of PMA, which had boosted IL-2 production induced by PHA, converted a negative result into a very robust positive one (8). The IL-2 response was highly specific for mAbs to T3 combined with PMA. Moreover, we found this result to be quite interesting: two stimuli, one putatively involving a component of the TCR complex, were required for the Jurkat line to produce IL-2.

We wondered how these two stimuli operated in concert to induce IL-2 production. Fortunately, we had an experienced molecular biologist in the lab, Bob Wiskocil, with whom to collaborate to address this question. Using Northern blot and dot blot analysis, Bob showed that the combination of PHA and PMA could induce the accumulation of abundant IL-2 transcripts in Jurkat, whereas PHA alone induced only low levels of IL-2 RNA (8). More interestingly, while neither OKT3 (an anti-T3 mAb) nor PMA induced detectable IL-2 RNA, the combination induced very robust accumulation of IL-2 transcripts. These results suggested that the two stimuli operated in concert to regulate the accumulation of IL-2 RNA.

Our results were consistent with work in the field involving complex mixtures of cells that suggested a two-signal requirement for IL-2 secretion or for T cell proliferation, but our studies simplified the study of this phenomenon by utilizing simple stimuli and a single cell type. Importantly, it provided a very simple experimental cell line model to study T cell activation of IL-2 production independently of T cell proliferation. Indeed, the Jurkat model, while having limitations, has proven extremely valuable as an experimental tool over the ensuing years with nearly 16,000 PubMed references for “Jurkat.” Numerous other studies have used Jurkat mutants to study signaling and other phenomena (9). Many of the proximal molecules involved in TCR signaling have been either discovered or validated in the Jurkat system. Moreover, our results suggested complex regulation for the IL-2 gene at the transcriptional level requiring multiple signal inputs, a notion that has since been well validated and expanded upon by the variety of signal inputs that have since been shown to regulate the IL-2 promoter and its 3 ′ untranslated region (10–12).

The observation that two stimuli could induce Jurkat to produce IL-2 validated the concept that stimulation of the TCR alone was insufficient to activate T cells. Dating back to 1970, Bretscher and Cohn had proposed that stimulation of the antigen receptor was insufficient to activate a naive T cell (13). Much of the early work on the two-signal model had confounded the field by the complex mixtures of cells used and the complexity of the antigens, second signals, and varied assays used to assess T cell activation. Our work provided Jurkat as a simplified T cell model, IL-2 RNA accumulation or secretion of this cytokine as a simple output for assessing activation and a simple stimulus for triggering the TCR. This simple model system supported the two-signal hypothesis and elaborated some of the integration of the two signals at the level of IL-2 gene regulation. The second signal was not well addressed by our paper in the *Journal of Immunology*. Instead of a physiologic stimulus, we used a small organic molecule, PMA, to provide the second signal. PMA had recently been shown to activate a protein kinase, protein kinase C (PKC) (14), which was later shown to consist of a family of kinases (15). It was also later shown that PMA also activates the Ras pathway by virtue of it ability to activate the guanine nucleotide exchanger RasGRP (16). Abundant work from many labs has well documented the critical roles for the activation of PKC and Ras in the pathways leading to IL-2 gene transcription. A short time after our *Journal of Immunology* paper discussed here, studies from our lab and from others identified mAbs to Tp44 (later named re-CD28) on Jurkat and on normal human T cells as being capable of delivering the critical second or “costimulatory” signal for the production of IL-2 (17, 18).

The reductionist approach toward studying T cell activation using the Jurkat line invigorated the lab and led to an exciting time of discovery that included not only studies of the two signal model of T cell activation, but also included: (1) the discoveries that stimulation of T3 or the TCR led to an intracellular calcium increase (19, 20); (2) the demonstration that the calcium increase in the cytoplasm was the result of activation of the inositol phospholipid pathway (21); and, (3) the discovery that the expression of the T3 complex required co-expression of the TCR αβ heterodimer (7, 22). During these exciting times, we were joined by several colleagues in the Stobo lab but most noteworthy were the contributions of John Imboden and Bob Wiskocil. We would also like to thank Kendall Smith for generously providing the Jurkat cell line that led to an incredible time of discovery and collegiality in the Stobo lab.

## Conflict of Interest Statement

The authors declare that the research was conducted in the absence of any commercial or financial relationships that could be construed as a potential conflict of interest.
